# Polypropylene Hollow Fiber Membrane by Dissolution-Inducing Pore Methods

**DOI:** 10.3390/membranes12050463

**Published:** 2022-04-25

**Authors:** Zhongyong Qiu, Chunju He

**Affiliations:** The State Key Laboratory for Modification of Chemical Fibers and Polymer Materials, College of Materials Science and Engineering, Donghua University, Shanghai 201620, China; chemist.qiu@outlook.com

**Keywords:** PP, ECMO, hollow fiber membrane, DIP

## Abstract

Plasma leakage limits the development of polypropylene membranes as oxygenated membranes. Here, a new method named the dissolution-induced pore method was adapted to prepare polypropylene hollow fiber membranes: after polypropylene and polyvinyl chloride were melt-blended and extruded, the polyvinyl chloride was removed by N, N-dimethylacetamide to obtain a porous polypropylene membrane material. The variation of membranes has been explored in detail with respect to the influence of different parameters on the flux and mechanical properties of membranes and the feasibility of the polyvinyl chloride recovery. The resulting polypropylene hollow fiber membrane shows that plasma penetration was zero within 6 h of test, gas flux can reach 189,000 L/(m^2^·h·0.1 MPa), and its strength at break reaches 65 MPa and the elongation at break is 890%; polyvinyl chloride recovery achieves more than 99%. This research has developed a promising and low-cost extracorporeal membrane oxygenation material, which provides benefits for patients with less capacity for medical expenditure.

## 1. Introduction

Extracorporeal membrane oxygenation (ECMO) is an effective means of treating critically ill cardiopulmonary dysfunction. ECMO has attracted much attention [1,2,3,4,5], as the coronavirus disease 2019 (COVID-19) continues to ravage the world since the use of ECMO is clinically beneficial for patients with severe heart failure or lung failure [6,7,8,9]. One of the key parts of ECMO comprises hollow fiber oxygenation membranes, which are made from polyolefin, e.g., polydimethylsiloxane, poly-1-methyl-4-pentene, polypropylene, and other polyolefin polymers [10], and polypropylene (PP) is one of the promising membrane material due to its stable, inexpensive, and easy-to-process characteristics.

Currently, there are two main methods to prepare polypropylene hollow fiber membranes (PPHFMs), i.e., thermally induced phase separation (TIPS) and melt stretching (MS) [11,12]. In the TIPS process, researchers explored the effect of diluent blended polymers, particles with hydrophilic groups [13,14,15], and PP grafted hydrophilic/hydrophobic segments on the performance of PP membranes [16]. Takahashi theorized that the water and gas fluxes of PPHFM for artificial lungs depend on the ratio of nucleating agent and temperature [17,18]. In the MS process, researchers studied the structural evolution of hollow membranes at different annealing temperatures [19], strength fields [20], and the effect of stretching ratio on pore size distribution [21]. Liu found that the extrusion rate and stretching temperature are the main factors for the gas flux of hollow fiber membrane as an artificial lung [22]. However, the limitations of TIPS and MS include pore diameters larger than 50 nm [23,24,25,26], which cannot meet the requirements of ECMO [27,28] for leading to plasma penetration [27,29,30,31,32], and this will pose a threat of infection to surrounding medical staff and cause dangerous biological pollution to the environment [33]. Therefore, it is necessary and of urgency to explore a new form of PPHFM without plasma leakage.

**Scheme 1:**
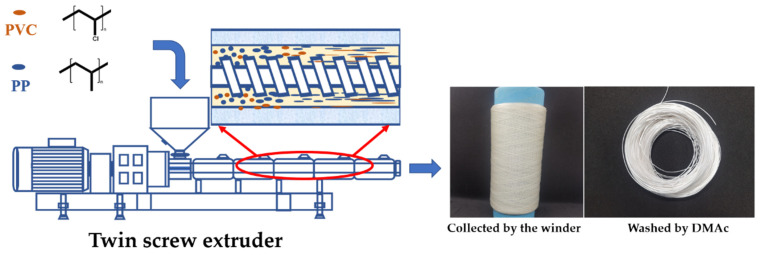
The illustration of prepared blend.

To solve the problem of plasma leakage of PPHFM, a new method, named dissolution-induced pore (DIP), was developed to prepare PPHFM for the first time. DIP refers to the method of melt blending of soluble polymer and matrix polymer via extrusion molding; then, soluble polymer was removed after solvent extraction, and the resulting membrane material offers permeable pores and adjustable structure. In this paper, polyvinyl chloride (PVC) and PP are blended to obtain a primary film, and then PVC is removed by solvent extraction to obtain a membrane (Scheme 1). Firstly, we prepared a series of flat membranes with different PVC contents with a flat vulcanizer, and we studied the flat membranes’ morphology, flux, and mechanical properties. Then, we prepared hollow fiber membranes with a twin-screw extruder and studied their morphology, flux, and biocompatibility. Finally, for environmental protection, we conducted a recycling study on the PVC.

## 2. Experimental

### 2.1. Materials

Polyvinyl chloride (PVC, Sinopharm Chemical Reagent Co., Ltd., Shanghai, China; molecular weight is two million) was dried at 70 °C for 5 h; polypropylene (PP, from Sinopec Co., Ltd., Shanghai, China, MI = 3.5 g) was used directly; and dimethylacetamide (DMAc, from Sinopharm Chemical Reagent Co., Ltd., Shanghai, China, chemically pure) was used directly without purification.

### 2.2. Preparation of Flat Membrane

It is a better choice to prepare a simple flat membrane to find the law before preparing the hollow fiber membrane. A certain amount of PP and PVC was melt-blended by the twin-screw extruder (TS-18/786, Huaju Co., Ltd., Nanjing, China) to obtain blended pellets. Then, the pellets were placed into a stainless mold at 190 °C with 10 MPa lasting 50 s with the flat vulcanizer (AL-01); then, the flat membranes were extracted with DMAc at room temperature for 12 h, cleaned with deionized water until it was solvent-free, and then dried for further tests. The membranes before and after extraction with DMAc were named *f-XXu* and *f-XX*, respectively, where *f* means flat membrane, *XX* means PVC content, and *u* means undissolved (Table 1).

### 2.3. Spinning of Hollow Fiber Membranes

The hollow fibers were spun with a twin screw at 190 °C, the winding stretch was 6 times, the winding speed was 6 m/s, the outer diameter of the fiber was controlled at 200–400 μm, and the wall thickness was 6–10 μm. After immersing the obtained hollow fiber in a DMAc solution at room temperature for 12 h, the hollow fiber membrane was cut and sampled for performance testing. The membranes before and after extraction with DMAc were named *h-XXu* and *h-XX*, respectively, where *h* means hollow fiber membrane (Table 2).

### 2.4. Membrane Performance

**Scheme 2:**
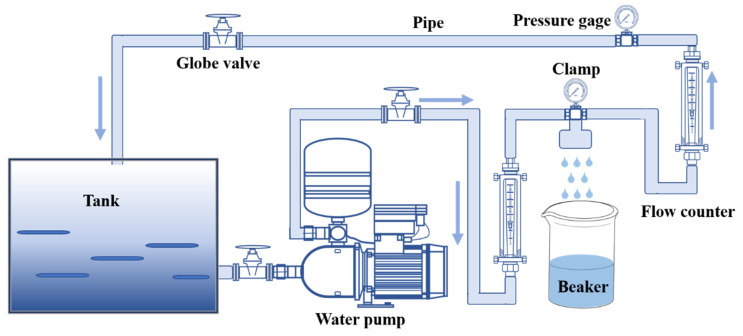
Water flux test device: the water is pressurized by the water pump to reach the membrane clamp, and the water pressure relative to the membrane is controlled by adjusting the water valves before and after the membrane clamp, and the flux is obtained by checking the difference between the flow meters before and after the membrane clamp.

**Scheme 3:**
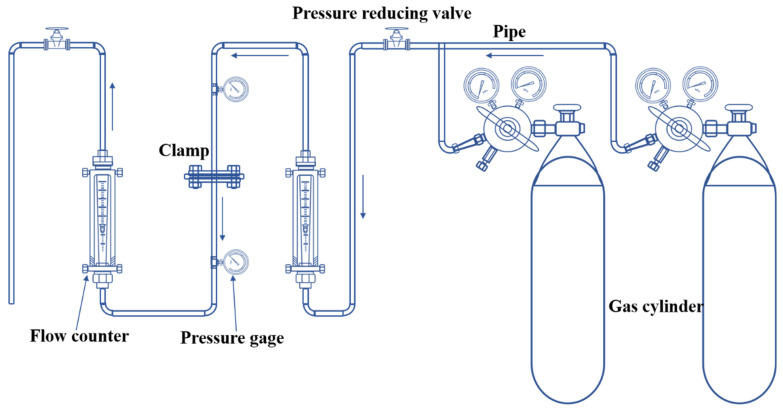
Air flux test device: the pressure value received by the membrane is controlled by adjusting the valve, and the gas flux is determined by the difference between the flow meters before and after the membrane clamp.

The flux test device (Scheme 2 and Scheme 3) developed by our group was used for the membrane’s flux test, as shown in Scheme 1 and Scheme 2, and flux (F) was calculated by Equation (1):

(1) $$F=\frac{V_{f}}{tPS}$$

where $V_{f\;}$(L) represents the volume of permeated water, P (0.1 MPa) is the pressure of the water during the test,  S (m^2^) is the area of membrane during filtration process, and t(h) is the duration time during filtering processes.

### 2.5. Membrane Porosity

The wetting method was used to test the porosity of membranes (*f-XX* and *h-XX*). The porosity ratio (Pr) is the volume ratio of air in the tangential volume of the membrane:

(2) $$\mathrm{Pr}=\frac{m_{1}-m_{0}}{\rho_{\mathrm{ethanol}}V_{0}}$$

where Pr (%) is porosity, $V_{0}$ (m^3^) is membrane volume, $m_{1}$ (kg) is mass of wet membrane,${\;m}_{0}$ (kg) is the mass of dry membrane, and ρ_ethanol_ (kg/m^3^) is the density of ethanol.

### 2.6. Mechanical Properties

Breaking elongation (Er) is the percentage of elongation at break, both ends of the fixed-length membranes are fixed on a clamp equipped with a tension sensor, and the pressure loaded on the membranes was collected by stretching at a constant speed until the membrane broke. The membranes (*f-XX* and *h-XX*) were tested by a stretching machine (Shenzhen Kaiqiang Co., Ltd., Shenzhen, China):

(3) $$\mathrm{Er}=\frac{\Delta L}{L_{0}}\times 100\%$$

where Er (%) is the breaking elongation, ΔL (cm) is difference between original length and breaking length, and $L_{0}$ (cm) is the original length of membrane.

The breaking strength (S) is calculated as follows:

(4) $$S=\frac{f}{S_{0}}$$

where S (Pa) is strength, f (N) is the force of the sample, and $S_{0}$ (m^2^) is the cross-sectional area of the sample.

### 2.7. SEM and FTIR Characterization

After being freeze-dried, the samples were embedded and cured with epoxy resin, and they were then broken in liquid nitrogen. The samples were then pasted on the stage with conductive glue for a 60s spray of gold, and scanning electron microscope (SEM, Hitachi’s SU8010, Minato-ku Tokyo, 1.5 kV, 10 μA) was used for observation. The Fourier Transform Infrared Spectrometer (FTIR, Nicolet iS50, Madison, WI, USA) was used to obtain molecular structure information.

### 2.8. Porogen Recovery

h-45u (m_2_/g) was placed into a container with DMAc (m_3_/g) at room temperature for t_1_ (h) hours; then, DMAc was recovered by distillation after the membrane was taken out, and the remaining (M/g) was obtained in a bottle from the container. The mass ratio of m_3_/m_2_ was set from 1 to 8 (t_1_ = 24). t_1_ was set from 2 to 16 (m_3_/m_2_ = 5). The theoretical content of PVC was M_0_ (M_0_ = m_2_ × 45%). The recovery rate (η) was calculated as follows.

(5) $$\eta =\frac{M}{M0}\times 100\%$$

### 2.9. Biocompatibility Test

The cell counting kit-8 (CCK8) method was adopted to detect the cytotoxicity of the membrane [34]. h-43 with 5 cm was sterilized with 75% alcohol for 5 min, then dried and irradiated with UV for 2 h, and h-43 was added in Dulbecco’s modified eagle medium [35,36] (DMEM, containing 10% fetal bovine serum and 1% double-antibody) at the ratio of 0.01 g/mL and 0.1 g/mL, and cultured in a 37 °C, 5% CO_2_ incubator for 24 h to obtain the extraction solution.

After 20 h, a cell was seeded in the 96-well plate with a density of 20 × 104 cells/mL containing a culture medium. The culture medium was discarded after the cells adhered to the wall, and 10%, 25%, 50%, and 100% of extraction solution were added, with 37 °C, 5% of CO_2_, and incubated for 1, 3, and 5 days, respectively. One hour before the end of the experiment, 10 microliters of CCK-8 were added per well and incubated in the dark. Absorbance was measured with a microplate reader (wavelength 450 nm).

## 3. Result and Discussion

### 3.1. Flat Membrane

#### 3.1.1. The Surface Morphology of Flat Membrane

**Figure 1:**
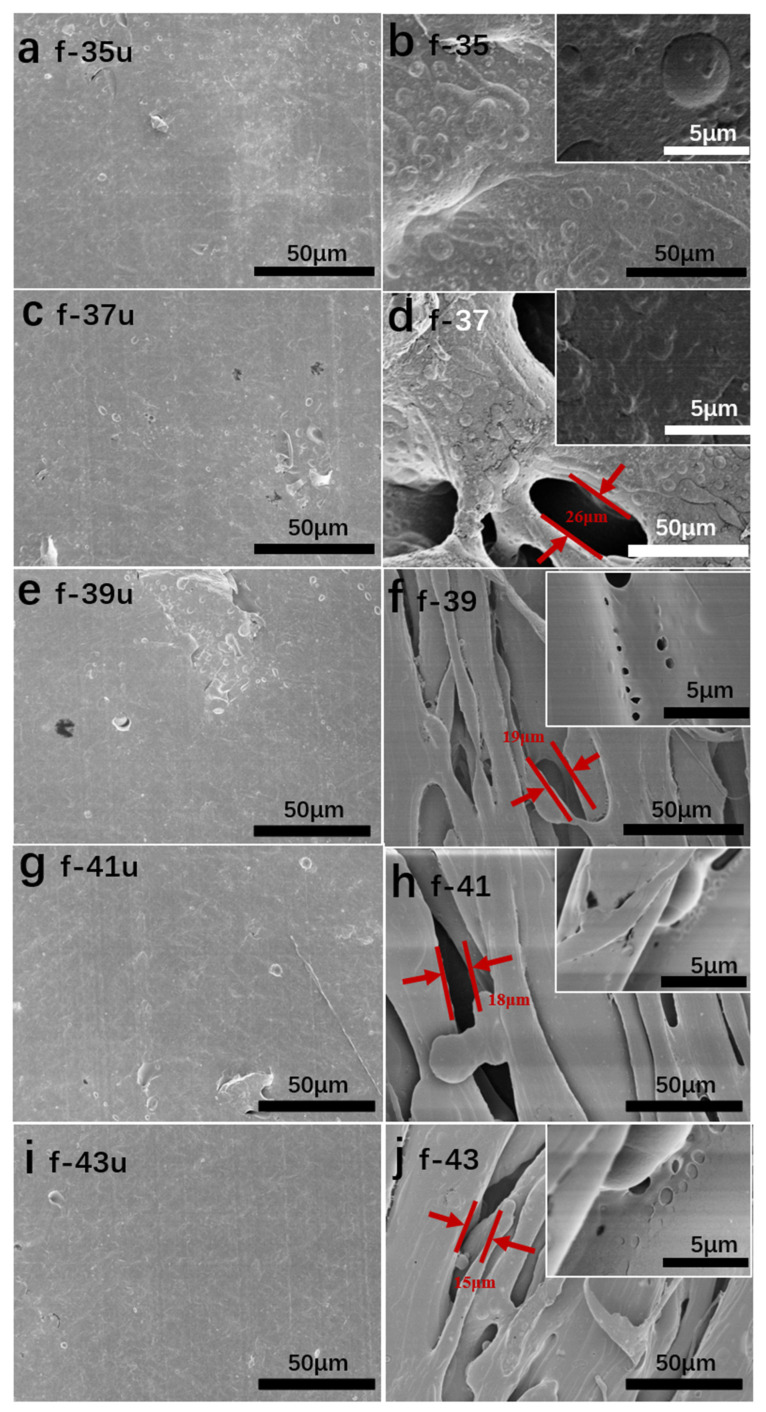
The surface morphologies of the flat membranes: (**a**,**c**,**e**,**g**,**i**) are the flat membranes without being washed by DMAc, and the contents of PVC are 35 wt.%, 37 wt.%, 39 wt.%, 41 wt.%, and 43 wt.%, respectively; (**b**,**d**,**f**,**h**,**j**) are the flat membranes washed by DMAc, and the contents of PVC are 35 wt.%, 37 wt.%, 39 wt.%, 41 wt.%, and 43 wt.%, respectively.

What is surprising is the obvious microfibril structure in Figure 1, and the pores of the membranes are not spongy pores or elongated pores in the general sense, which is the gaps between microfibrils, and this is also a feature of the DIP method. The pore size of membranes has an increasing tendency with the increase in PVC content. When the content of PVC is 35 wt.%, there are no pores or gaps on the rough surface of the membrane. The gaps were observed when the content of PVC was 37 wt.%. Moreover, dendritic microfibers appear when the PVC content is 39 wt.%, the microfibers are bifurcated and overlapped with gaps, and the adhesion between the microfibers maintains the basic shape of the membrane. Microfibril is the most notable feature of the flat membrane with DIP, which is formed by the blending and stretching processes of the PP and PVC [37,38,39,40]. Microfibrillation usually occurs during the melt blending and orientation of incompatible two-phase polymers [41,42].

#### 3.1.2. The Flux of Flat Membranes

**Figure 2:**
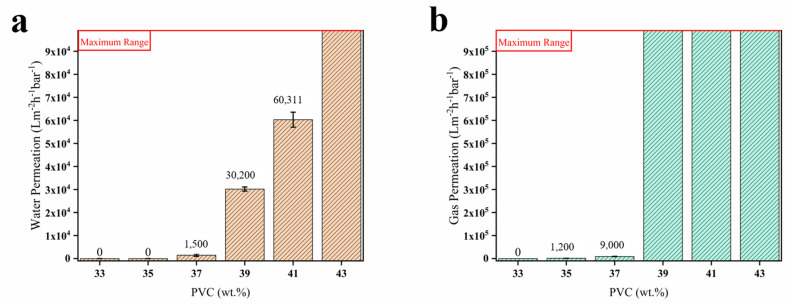
Water flux (**a**) and air flux (**b**) of flat membranes.

As shown in Figure 2, when PVC content is 33 wt.%, both the water and air fluxes of the flat membranes are 0 L/(m^2^·h·0.1 MPa). With the increase in PVC content, the growth rates of water flux and air flux are significantly different since the increasing rate of gas flux is significantly greater than that of water flux. When PVC content is 35 wt.%, the water flux is 0 L/(m^2^·h·0.1 MPa), while the air flux is 1200 L/(m^2^·h·0.1 MPa). When PVC content is 39 wt.%, the water flux reaches 30,200 L/(m^2^·h·0.1 MPa), and the air flux is greater than 1,000,000 L/(m^2^·h·0.1 MPa). When PVC content is further increased to 43 wt.%, the water flux exceeded the maximum range of the equipment, which means that water flux is greater than or equal to 100,000 L/(m^2^·h·0.1 MPa). The content of PVC can directly control the filtration performance of the flat membrane, and this is also an advantage of the DIP method. The increase in pores inside the membrane for water or gas to flow through is an indirect cause of the increase in gas flux and water flux [43]. The fundamental reason is that the increase in PVC content leads to an increase in the volume of the blend that can be dissolved by DMAc, and porosity increased after PVC was dissolved.

#### 3.1.3. The Mechanical Properties of Flat Membranes

**Figure 3:**
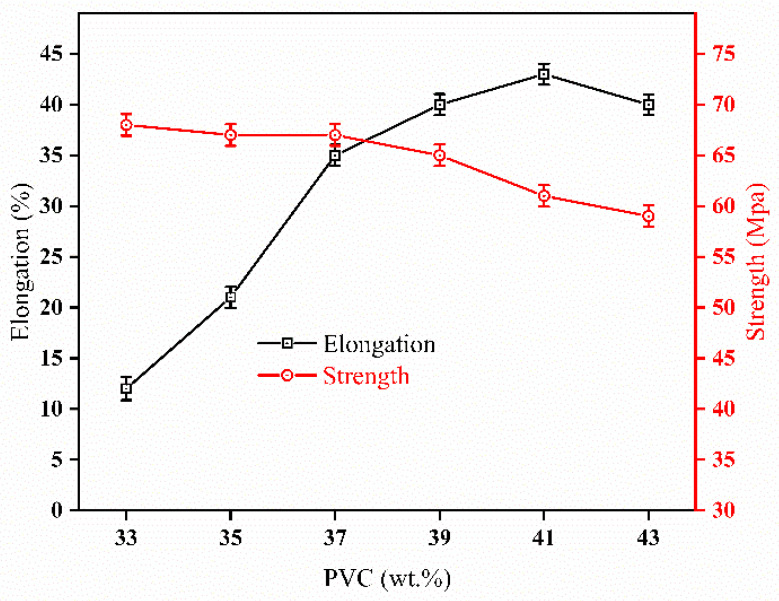
Elongation at break and strength of flat membranes.

The strength of membranes continuously declines as the PVC content continuously increased (Figure 3), which decreased from 68 MPa to 58 MPa as the PVC increased from 33 wt.% to 41 wt.%, indicating that the increase in PVC content can reduce the flat membrane’s strength properties. However, the elongations at break and PVC content both have an upward trend, and the elongation at break increased from 42% to 71%, which is the maximum value. This interesting change may be due to the oriented microfibers that improved elasticity and reduced strength. Because microfibers have good internal macromolecular orientation [44], which will increase the elongation at the break of the membrane, and the degree of microfibrillation is determined by the PVC content, when the PVC content increases, the PP content will decrease. Then, by decreasing the number of microfibers, a smaller number of microfibers will directly reduce the membrane’s strength.

#### 3.1.4. The Porosity of Flat Membranes

**Figure 4:**
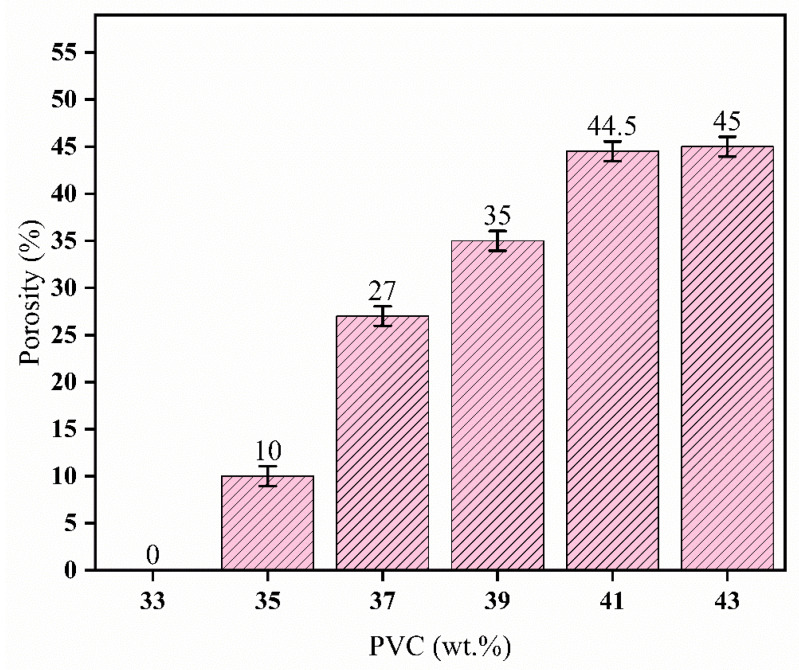
Porosity of flat membranes.

As shown in Figure 4, there is an obvious positive correlation between porosity and PVC content. In particular, the porosity of the flat membrane is 0% when the PVC content is 33 wt.%; therefore, most of the PVC is completely wrapped by PP, and it is difficult to contact DMAc. When the PVC content is 41 wt.%, the porosity of the membrane is 44.5%, which is larger than PVC content. Thus, PVC content can directly control porosity, and it once again shows the advantage of the DIP method to directly control the soluble content in order to control membrane performance, because the change of porosity will directly affect the permeation performance of the membrane. As porosity increases, the volume that can supply water or gas to pass through will increase, which will directly increase the flux of the membrane. This is proved in the results of Figure 2, and the main factor affecting porosity is PVC content.

### 3.2. Hollow Fiber Membrane

#### 3.2.1. The Morphology of Hollow Fiber Membranes

**Figure 5:**
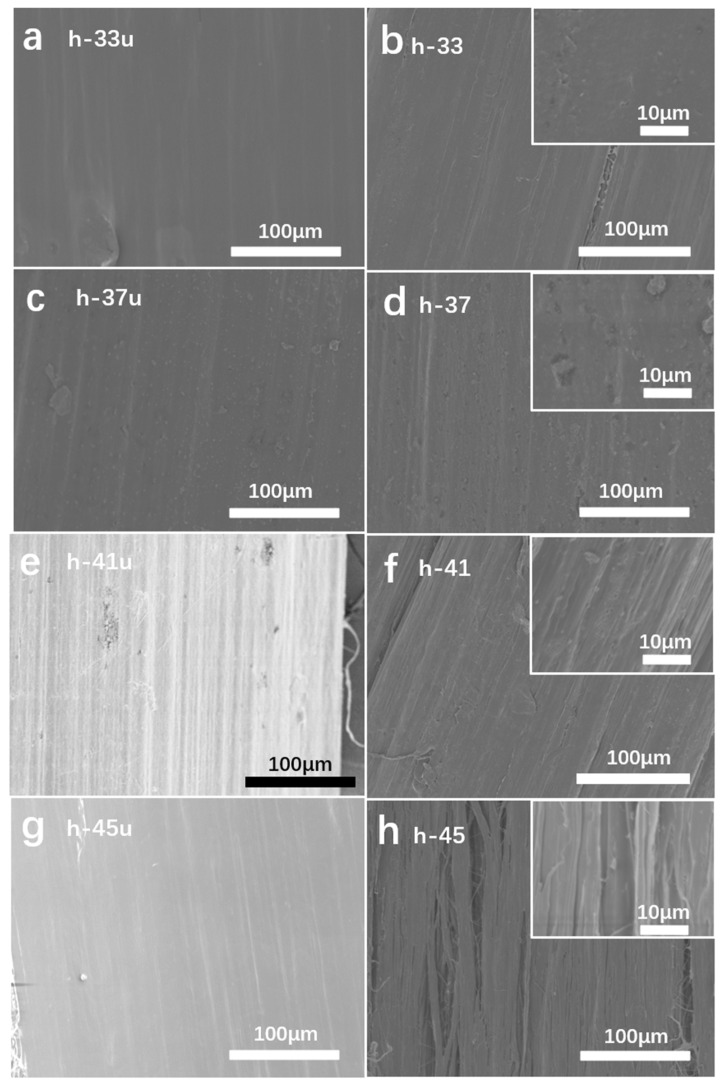
The surface morphologies of hollow fiber membranes: (**a**,**c**,**e**,**g**) are the hollow fiber membranes without being washed by DMAc, and the contents of PVC are 33 wt.%, 37 wt.%, 41 wt.%, and 45 wt.%, respectively; (**b**,**d**,**f**,**h**) are the hollow fiber membranes washed by DMAc, and the contents of PVC are 33 wt.%, 37 wt.%, 41 wt.%, and 45 wt.%, respectively.

Compared with the flat membrane (Figure 1), the morphologies of the hollow fiber membranes (Figure 5) have commonalities and differences. The commonalities are microfibrillation and the morphology trend with the change of PVC content, which are the same as the flat membranes, so it will not be repeated here. The obvious difference is the size of the microfibers of hollow fiber membrane, which is thinner than the flat membrane due to the larger stretch ratio. This morphology is unique to the DIP method in the formation method of the membrane material as far as we know.

#### 3.2.2. The Flux of Hollow Fiber Membranes

**Figure 6:**
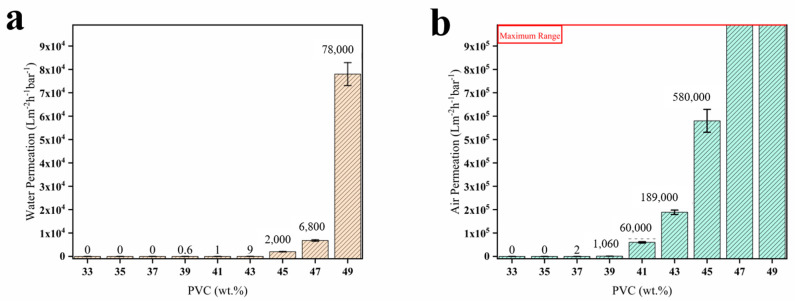
Water flux (**a**) and air flux (**b**) of hollow fiber membranes.

Although the water flux and air flux of hollow fiber membrane have a general increasing trend with the increase in PVC content (Figure 6), the initial growth point and the growing range are different. When PVC content is less than 37 wt.%, the water flux and air flux are zero. When PVC content reaches 37 wt.%, gas flux begins to appear, i.e., 2 L/(m^2^·h·0.1 MPa), while the water flux is 0 L/(m^2^·h·0.1 MPa). When PVC content reaches 39 wt.%, the water flux is 0.6 L/(m^2^·h·0.1 MPa) and the air flux is 1060 L/(m^2^·h·0.1 MPa). As PVC content increased from 41 wt.% to 43 wt.%, the water flux increases from 1 L/(m^2^·h·0.1 MPa) to 9 L/(m^2^·h·0.1 MPa), and the air flux increases from 60,000 L/(m^2^·h·0.1 MPa) to 189,000 L/(m^2^·h·0.1 MPa). This is a delightful line of data points, with extremely high air flux and low water flux, and it is an ideal candidate for ECMO membranes.

#### 3.2.3. The Mechanical Properties of Hollow Fiber Membranes

**Figure 7:**
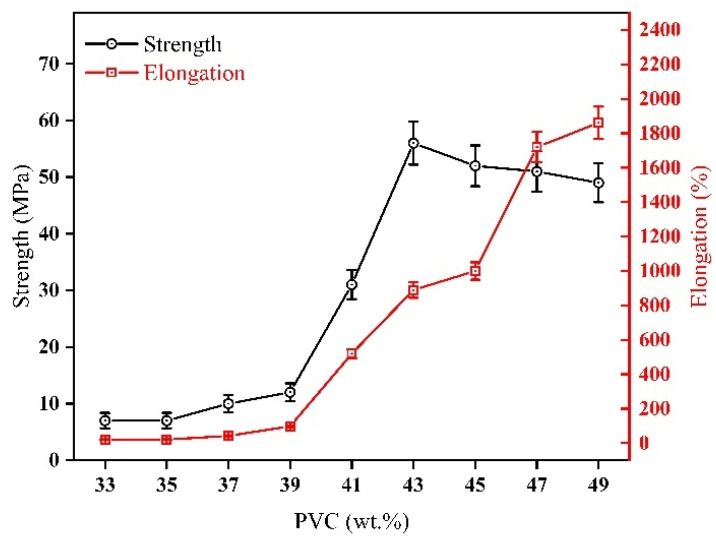
The elongation at break and strength of hollow fiber membranes.

As observed in Figure 7, with the increase in PVC content, the strength shows a tendency to rise and then fall; when the PVC content is less than 39%, the strength increases slowly as the PVC content increases. When the PVC content increases from 39 wt.% to 43 wt.%, the strength rapidly reaches the maximum value of 56 MPa from 11 MPa. Elongation at break increases with increasing PVC content. The PVC content is the main factor affecting the mechanical properties of the membrane. Compared with the flat membrane, the elongation at the break of the hollow fiber membrane is higher and the strength is smaller. The main difference between the two in structure is the size of the microfiber. The smaller size of the microfiber is helpful for improving elongation. At the same time, stretching is a key factor in the formation of microfibers of hollow fiber membranes.

#### 3.2.4. The Porosity of Hollow Fiber Membranes

**Figure 8:**
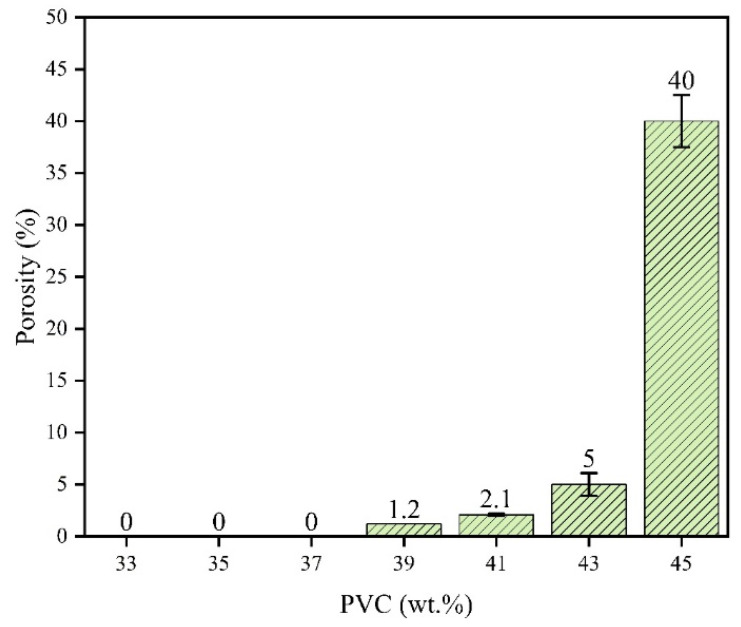
The porosity of hollow fiber membranes.

The porosity of the membrane tends to increase with the increase in PVC content (Figure 8). The porosity of membranes is less than 3%, which also explains well that the water flux is not greater than 1 L/(m^2^·h·0.1 MPa) when PVC content is less than 41 wt.%. Porosity presents an obvious increasing trend with increasing PVC content from 41 wt.% to 45 wt.%, and porosity increased from 0.5% to 40%. In particular, when the PVC content is 45 wt.%, the porosity is 40%, which is eighttimes the membrane with 43 wt.% PVC content. The porosity of the membrane is regulated by PVC content.

### 3.3. The Biocompatibility of Hollow Fiber Membranes

**Figure 9:**
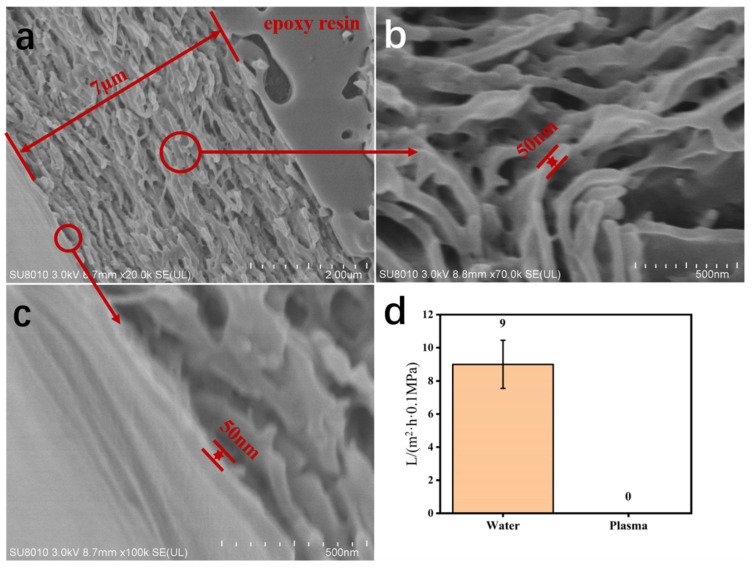
(**a**–**c**) Cross-section of h-43. (**d**) The water flux and plasma flux of h-43.

Figure 9 shows the morphology of the cross-section, the cross-section of h-43 presents the characteristics of a thinner wall with a loose main structure, and the main structure in the form of a skeleton structure can ensure strength and, at the same time, induces minimal resistance to the gas mass transfer process. In addition, the thickness of the skeleton in the structure is about 50 nm. The wall thickness of the hollow fiber membrane is about 50 nm and is relatively uniform without obvious defects or breakage. The result indicates that the thinner wall thickness still has a certain strength to maintain the basic shape and function. The thin wall of the hollow fiber membrane is the functional layer that mainly blocks the liquid from passing through the gas. The thinner functional layer can reduce gas mass transfer resistance [45]. However, the separation layer here is thin, which induces a risk of water permeability. h-43 shows excellent waterproof and gas performance, and the direct evidence is that no plasma leakage was found in the long-term plasma permeation experiment (Figure 9d). Table 3 shows the flux comparison results of polypropylene oxygenation membranes. Our DIP method polypropylene oxygenation membrane has better performance in blocking plasma permeability, which fully shows the great potential of the emerging DIP method.

**Figure 10:**
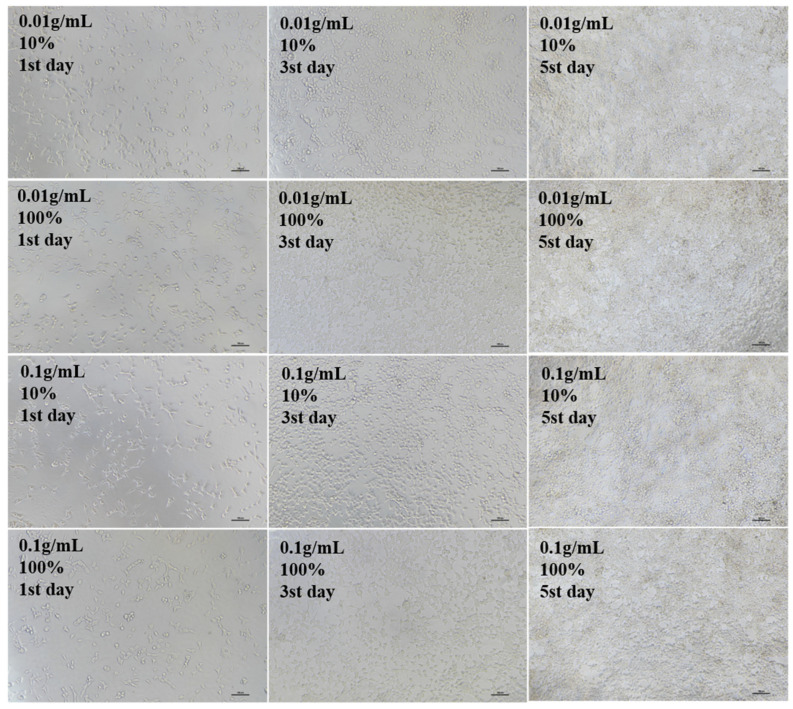
Human-derived 293T cells’ pictures of different concentrations of extract and different incubation times.

Figure 10 shows the results of the cytotoxicity comparison test, in which the cell densities for the same incubation time did not show significant differences. One-way ANOVA statistical analysis was used [46], and there was no difference in the absorbance values at 450 nm between the experimental group and the control group, and this result suggests that the material does not affect the proliferation of human-derived 293T cells. That is, in vitro experiments demonstrated that h-43 has good biocompatibility for ECMO. The PP cytotoxicity test has many studies and confirmed conclusions [47]; moreover, PP as a commonly used material in direct contact with cells, has not been reported to exude toxins [48], and we obtained positive results with respect to cytotoxicity, which means that the membrane material is safe, and in reverse, the material that makes up the membrane is safe.

### 3.4. Porogen Recovery

**Figure 11:**
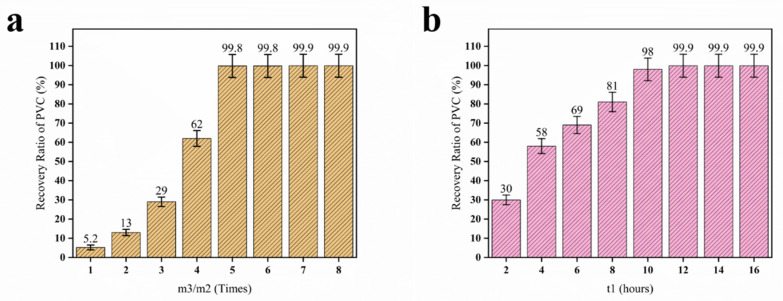
The relationship between the mass ratio of m_3_/m_2_ (DMAc/PVC) and η (recovery rate of PVC) at room temperature for 24 h (**a**); the relationship between the extraction time and η (**b**).

It is necessary to recycle PVC to reduce costs and emissions. As shown in Figure 11, when the ratio of DMAc/PVC is less than 5, the recovery rate increases with the increase in m_3_/m_2_; when m_3_/m_2_ is bigger than 5, the recovery rate reaches 99.8% and remains unchanged with further increases in DMAc/PVC. The recovery ratio shows an increasing trend with the increase in extraction time, which reaches 99.9% after 12 h of extraction and does not change with further increasing extraction time. Therefore, the best condition for the recovery of PVC is the extraction of 12 h when the weight of DMAc is five times the weight of PVC. Therefore, the DIP method that we present here is an unreported process that is recyclable.

**Figure 12:**
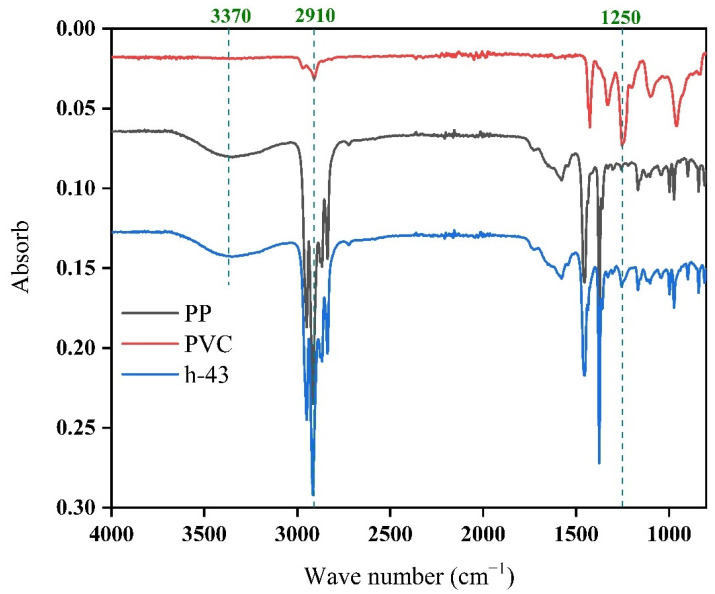
The FTIR of h-43.

Figure 12 shows the infrared spectrum of membrane h-43, which clearly shows that h-43 has a strong absorption peak of methyl hydrogen (2910 cm^−1^, ν_as_), which is consistent with PP materials. The ethyl group in PVC also has a moderate absorption peak here, which is also consistent with the structure of PVC. Among the infrared absorption peaks of h-43, the characteristic absorption peaks observed everywhere are consistent with PP, indicating that most PVC in h-43 has been removed, which was confirmed by the porogen recovery experimental result where 99.9% PVC was removed. Moreover, the residual PVC is 0.1%. In the above biocompatibility test, h-43 showed good biocompatibility, which meets the biocompatibility requirements of ECMO.

## 4. Conclusions

A dissolution-induced pore method was explored to prepare a PP membrane with connected structure via melt blending of PP and PVC. The morphology and flux of the resulting membrane can be controlled by the content of PVC easily, and PVC can be recycled. The PP hollow fiber membrane shows excellent air permeability, water barrier properties, and biocompatibility; the air flux reaches 189,000 L/(m^2^·h·0.1 MPa) with no plasma penetration and fits the basic requirement of ECMO. It also provides a promising material for the supply of medical oxygen for the membrane. It has great potential in clinical applications and provides the possibility to treat critical patients.

## Figures and Tables

**Table 1:** Flat membrane.

Name (before Washed by DMAc)	Name (after Washed by DMAc)	PP wt.%	PVC wt.%
f-33u	f-33	67	33
f-35u	f-35	65	35
f-37u	f-37	63	37
f-39u	f-39	61	39
f-41u	f-41	59	41
f-43u	f-43	57	43

**Table 2:** Hollow fiber membrane.

Name (before Washed by DMAc)	Name (after Washed by DMAc)	PP wt.%	PVC wt.%
h-33u	h-33	67	33
h-35u	h-35	65	35
h-37u	h-37	63	37
h-39u	h-39	61	39
h-41u	h-41	59	41
h-43u	h-43	57	43
h-45u	h-45	55	45

**Table 3:** Oxygenation membrane performance comparison.

Name	Water Flux	Air Flux	Plasma Flux	From
EC-1	1.1 mL/(min·cm^2^·atm)	68 mL/(min·cm^2^·atm)	0.191 mL/(min·cm^2^·atm)	[17]
None	5.74 mL/(min·cm^2^·atm)	300 mL/(min·cm^2^·atm)	0.587 mL/(min·cm^2^·atm)	[17]
80 (Water)	-	0 L/(min·m^2^·atm)	-	[18]
60 (Freon)	-	17 L/(min·m^2^·atm)	-	[18]
120 (Paraffin)	-	416 L/(min·m^2^·atm)	-	[18]
120 (PEG200)	-	432 L/(min·m^2^·atm)	-	[18]
120 (PEG300)	-	367 L/(min·m^2^·atm)	-	[18]
120 (SH200)	-	361 L/(min·m^2^·atm)	-	[18]
h-43	9 L/(m^2^·h·0.1 MPa)	189,000 L/(m^2^·h·0.1 MPa)	0 L/(m^2^·h·0.1 MPa)	This study

## Data Availability

Not applicable.
